# Perforated mucocele of the appendix in the left upper quadrant: A challenging anatomy and an improvised surgical technique

**DOI:** 10.1016/j.ijscr.2019.05.020

**Published:** 2019-05-11

**Authors:** Basmah Faris Alhassan, Abdullah Saji Alharbi, Walid Mokhtar Omar, Mohammed Ayesh Zayed, Maha Abdulla, Thamer Abdulla Bin Traiki

**Affiliations:** a Department of General Surgery, King Khalid University Hospital and College of Medicine, King Saud University, Saudi Arabia; b Department of Anesthesia, King Khalid University Hospital and College of Medicine, King Saud University, Saudi Arabia; c Department of Radiology, King Khalid University Hospital and College of Medicine, King Saud University, Saudi Arabia; d Colorectal Research Chair, Department of General Surgery, King Khalid University Hospital and College of Medicine, King Saud University, Saudi Arabia

**Keywords:** Case report, Pseudomyxoma peritonei, Mucocele, Malrotation, Colon resection, HIPEC

## Abstract

**Introduction:**

Pseudomyxoma peritonei (PMP) is a feared complication of appendicular mucocele perforation. Although a rare disease, its major sequel mandates recognition and early intervention. Intestinal malrotation is mostly asymptomatic in adults. Its significance arises when it complicates another coinciding condition by confusing the presentation, leading to delay in diagnosis and treatment. PMP and incidental finding of gut malrotation in adults are two rare events, and the chance of both occurring in the same patient is very slim. This can complicate the clinical picture and lead to devastating outcomes.

**Presentation of the case:**

We present a case of PMP in a patient with gut malrotation, managed with cytoreductive surgery (CRS) and heated intraperitoneal chemotherapy (HIPEC).

**Discussion:**

Management with CRS/HIPEC has been found to improve outcomes for patients with PMP. However, the extensive disease and abnormal anatomy of the patient in our report proposed unique intraoperative challenges. Preserving part of the colon was possible with an improvised surgical technique that we used which proved to be safe and effective.

**Conclusion:**

Early recognition and consideration of uncommon but serious surgical conditions are essential for improved patient outcomes. To our knowledge, this is the first report in the English literature that describes the use of CRS/HIPEC for PMP in a case of intestinal malrotation. This improvised surgical technique was found to be safe and can provide a surgical solution for preserving part of the colon in selected patients.

## Highlights

• Pseudomyxoma peritonei (PMP) is a devastating consequence of perforated appendicular mucocele. It is considered a rare disease.
• The presence of asymptomatic gut malrotation in adults can complicate the clinical picture of acute abdomen. ** (starting a new highlight point): Not considering possible anatomical variations initially during assessment could delay the diagnosis and managment.
• The introduction of Cytoreductive Surgery/Heated Intraperitoneal Chemotherapy (CRS/HIPEC) improved the outcome of many patients with PMP.
• Preserving part of the colon during challenging colorectal resection to allow a colo-rectal anastomosis offers a superior quality of life.

## 1 Introduction

Mucocele of the appendix is considered a rare disease. Presentation can vary and can be misleading [1]. This is even more pronounced when an underlying malrotation is present because this creates a confusing clinical picture. The unclear clinical picture can lead to a significant delay in diagnosis and management, which can in turn result in perforation and the devastating sequel of pseudomyxoma peritonei (PMP).

The Peritoneal Surface Oncology Group International (PSOGI) defines PMP as the “intraperitoneal accumulation of mucus due to mucinous neoplasia characterized by the redistribution phenomenon. It can include mucinous ascites, peritoneal implants, omental cake” [2]. Classifying PMP has been controversial. The most recent consensus reached by the PSOGI in 2016 proposed that PMP can be classified into the following three categories: 1) low-grade mucinous carcinoma peritonei (or low-grade appendicular mucinous neoplasm [LAMN]); 2) high-grade mucinous carcinoma peritonei; 3) high-grade mucinous carcinoma peritonei with signet ring cells [2]. Regardless of the category, PMP of the appendix has long been considered a lethal and terminal disease. However, survival rates and patient outcomes dramatically improved after the introduction of management with cytoreductive surgery (CRS) and heated intraperitoneal chemotherapy (HIPEC) [3,4].

We report a case of PMP with abnormal rotation of the intestines where the appendix lies in the left upper quadrant (LUQ). The patient was managed with CRS/HIPEC. This seems to be the first case of PMP accompanied by such a constellation of unique challenges to be reported in the English literature. We also describe the improvised surgical technique we applied that can be used to overcome some of the challenges presented in this case report.

This case report was written following the Surgical CAse REport (SCARE) guidelines [5], and is registered as a first-in-man study on the Research Registry website (www.researchregistry.com) as UIN 4485.

## 2 Patient presentation

**Fig. 1:**
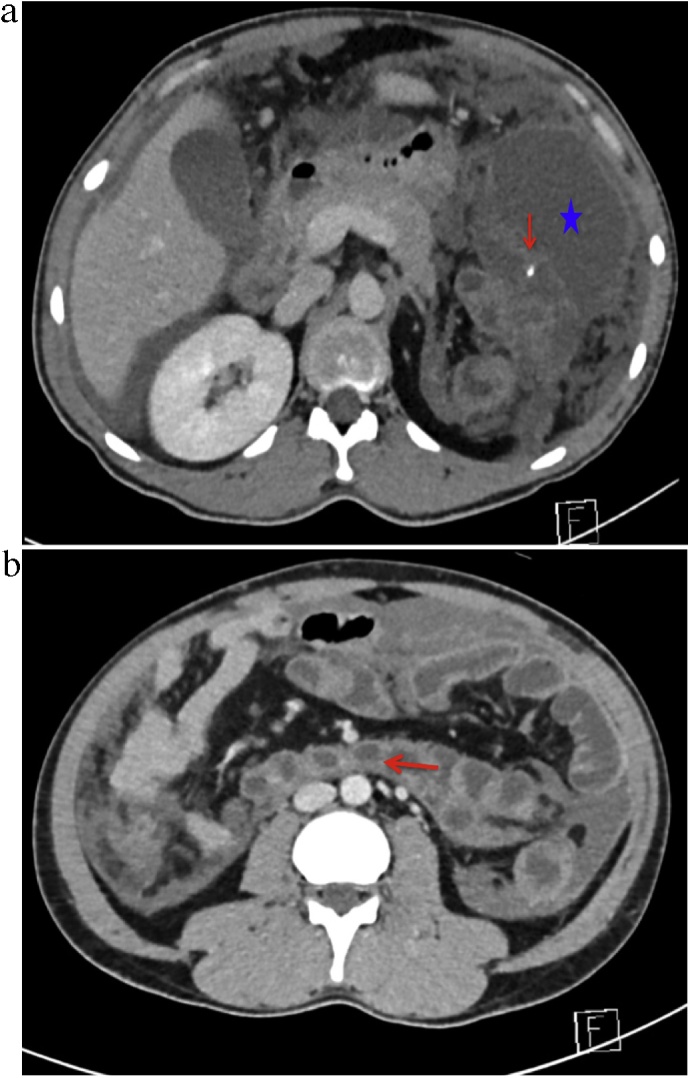
Pre-operative CT images: **A)** Large phlegmon in LUQ “star” with intra-luminal calcification “arrow”. A large right kidney is also noted. **B)** Note part of the ascending colon “arrow” is running behind the root of mesentery.

The patient is a 36-year-old male with no significant past medical history. He presented to a local hospital with a complaint of left-sided abdominal pain for several days. He was initially managed conservatively as a non-specific abdominal pain and discharged home. Two months later, he re-presented with severe abdominal pain associated with signs and symptoms of sepsis. Computed tomography (CT) scanning of the abdomen revealed a phlegmon in the LUQ and free fluid (Fig. 1A). A diagnostic laparoscopy was performed, and showed extensive peritoneal deposits of thick mucoid material. Biopsies were taken, washout was performed, and drains were inserted. Later, his sepsis resolved, and the biopsies showed LAMN.

He was then referred to our hospital for further management. Upon evaluation, upper and lower gastrointestinal scopes were performed and showed no evidence of pathology.

Reviewing the CT imaging identified the abnormal anatomy of the bowel where the right colon runs retroperitoneally behind the mesentery of the small bowel. The cecum with the appendicular mass was found in the left side of the abdomen joining the colon at the splenic flexure. The duodenojejunal (D–J) junction was found slightly to the right side of the midline, and the inferior mesenteric vein to its left side. In addition, a large right kidney, an atrophied left kidney, and two ureters were noted (Fig. 1B).

The case was discussed in a multidisciplinary meeting (Tumor Board), where the committee advised to proceed management with CRS/HIPEC.

**Fig. 2:**
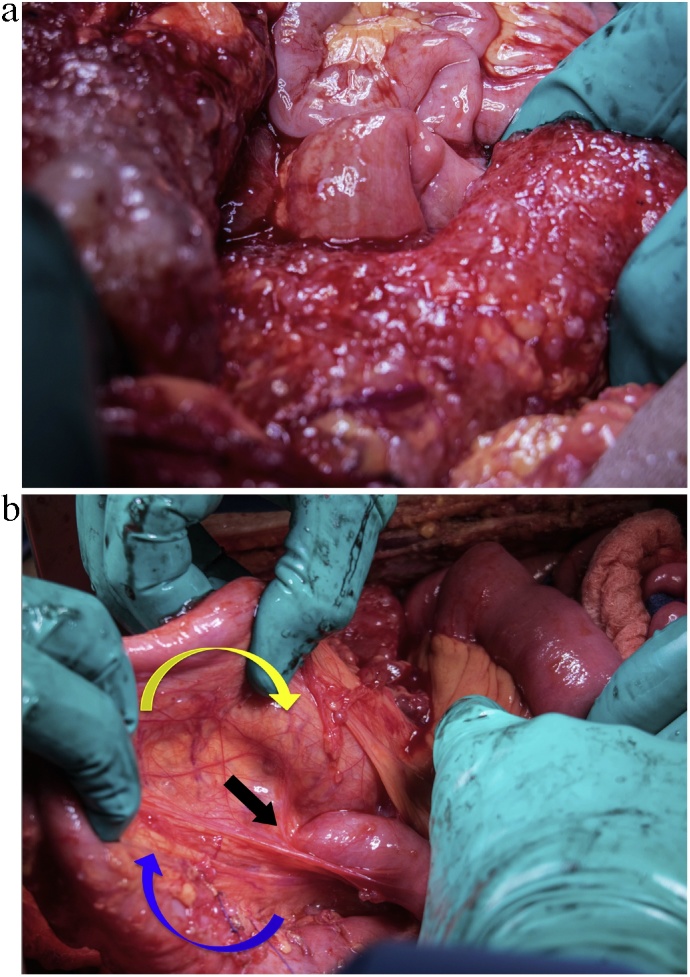
**A)** Extensive peritoneal disease with carpet-like thick mucoid deposits. **B)** The D–J junction “black arrow” is right to the midline. The ascending colon “blue arrow” is coming partially behind the root of mesentery and then continues anteriorly as the transverse colon “yellow arrow”.

**Fig. 3:**
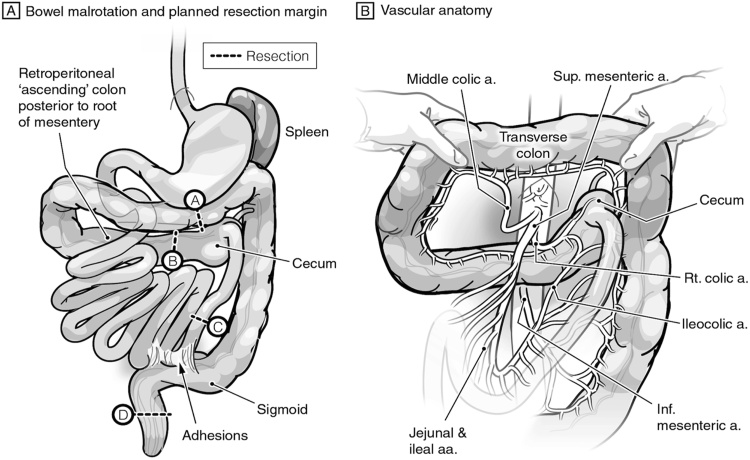
The abnormal anatomy of the gut: **A)** Schematic representation of the malrotation margins of the en-bloc resection delineated with markings “- - - -”. The A–D labeling to facilitate follow up on coming figures. **B)** Another view with the transverse colon elevated upwards to reveal the vascular anatomy. a: artery. Sup: Superior. Rt: Right. Inf: inferior.

Exploratory laparotomy revealed extensive deposits all over (Fig. 2A), giving a Peritoneal Cancer Index (PCI) of 39. The abnormal anatomy was clarified as described in the CT scan. The duodenum was partially intraperitoneal (Fig. 2B). There were congenital adhesions between the sigmoid colon and the small bowel mesentery. Severe adhesions were also apparent in the LUQ—phlegmon-like—involving the greater omentum, cecum, appendix, splenic flexure and spleen. This phlegmon was resected en-bloc to include the spleen, greater omentum, terminal ileum, and part of the ascending, distal transverse, and splenic flexure colon. However, most of the left colon, sigmoid, and the intraperitoneal rectum were also heavily diseased. Thus, the resection was extended to include the entire colon beyond the distal transverse with the upper two-thirds of the rectum (Fig. 3A and B). Complete cytoreduction was achieved through stripping the entire peritoneum, including the peritoneal lining of the diaphragm, burning the liver surface, and resecting both the greater and lesser omentum. In addition, small bowel mesentery and its surface were cleared from the disease.

**Fig. 4:**
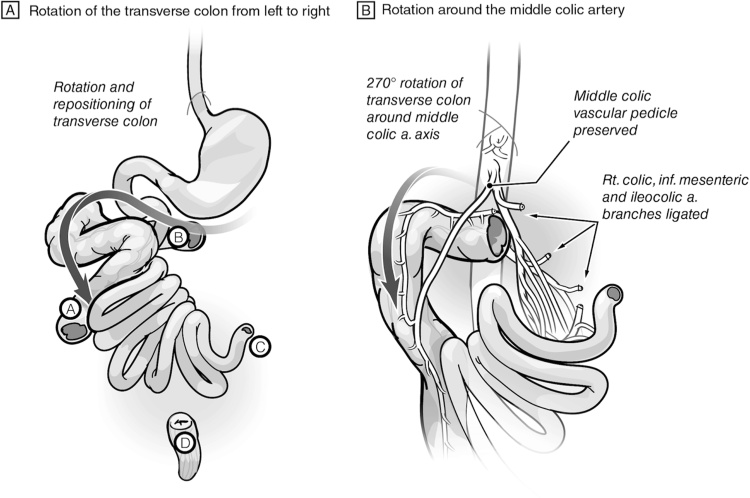
The improvised technique: **A)** After the en-bloc resection, the distal end of the transverse colon rotated from left to right and repositioned to reach the low rectum along the right side. **B)** The rotation of the transverse colon involves the 270^0^ anti-clockwise rotation around the middle vascular pedicle. a: artery. inf: inferior.

Creating a tension-free anastomosis with good vascularity between the remaining transverse colon and the low rectum constituted a great challenge. The segment was dependent on the middle colic pedicle, and could not reach to the low rectum in the current anatomical position. Considering this unusual challenge, we aimed to preserve the transverse colon by rotating the colon along the axis of the middle colic pedicle from left to right after scoring the peritoneal surface of the mesentery. At this position, the distal end of the transverse colon reached the low rectum without tension through the right side (Fig. 4A and B).

Given this procedure has not been described before, we questioned the perfusion and tension in that new position. Therefore, the colon was left in the proposed position for 90 minutes, during which we performed the HIPEC by infusing heated intraperitoneal mitomycin C and doxorubicin, in addition to intravenous 5-fluorouracil and leucovorin.

**Fig. 5:**
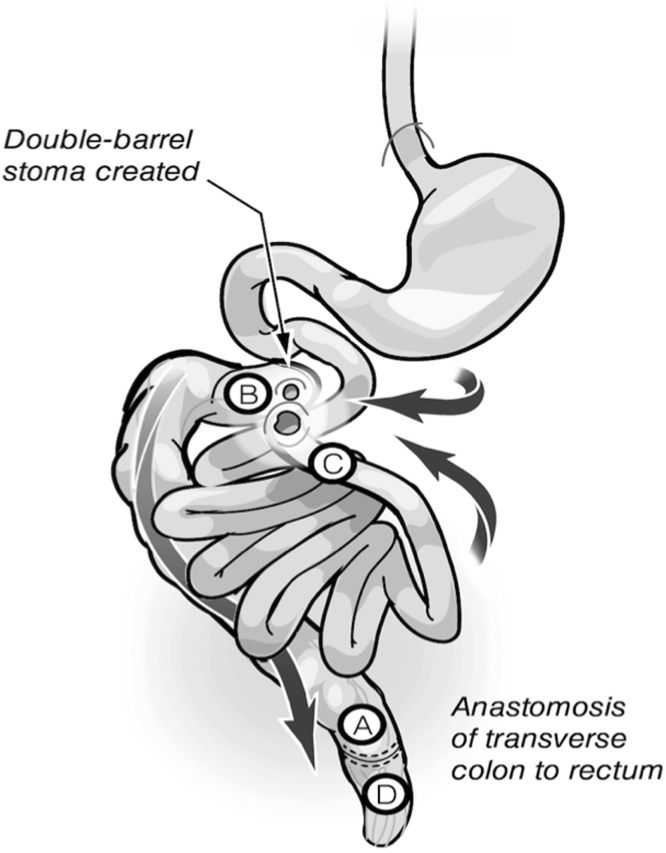
The final anatomy after re-construction: double barrel stoma created with proximal end of the transverse colon and ileum. The rotated end of the transverse colon is anastomosed to the low rectum in the new anatomical position.

Upon completion of HIPEC, the colon retained normal vascularity and viability in the new rotated position. Therefore, we proceeded with the anastomosis by connecting the low rectal stump to the rotated distal transverse colon, using the stapled technique (Fig. 5). After washing the abdominal cavity with warm water and re-inspecting the bowel, drains were placed intraabdominally and bilateral chest tubes were inserted. The midline laparotomy wound was closed, and a double-barrel stoma was created with the distal ileum as one end, and the proximal part of the remaining colon as the mucous fistula (Fig. 5).

**Fig. 6:**
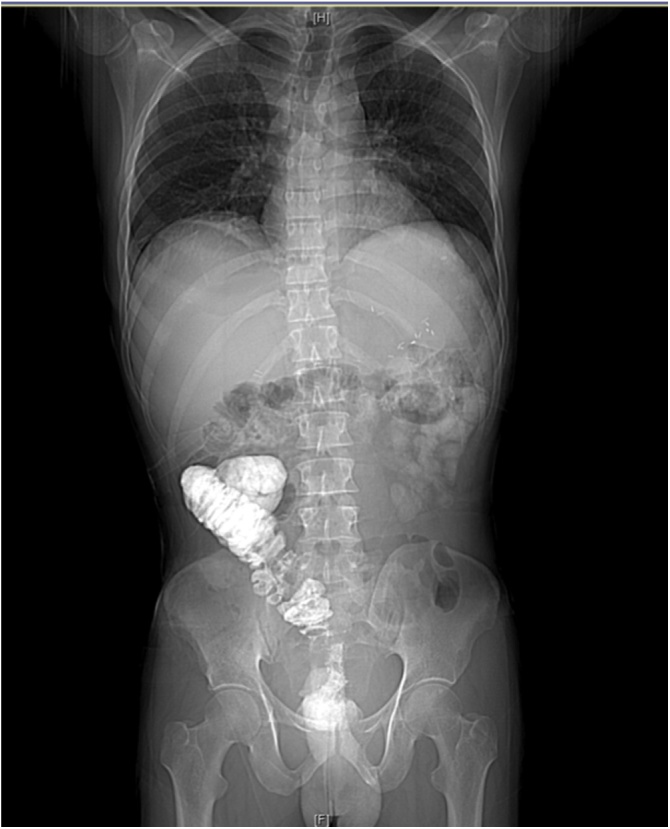
Post operative Barium study. The flowing of contrast through the stoma demonstrates the new anatomy.

The patient was extubated at the end of the procedure, and transferred to the intensive care unit (ICU), where he recovered gradually for several days, and was then transferred to the surgical ward. Heparin infusion was initiated eight hours postoperatively as an attempt to protect against a concerning risk of thrombosis in the rotated vascular pedicle, which could endanger the blood supply to the remaining colon. Unfortunately, during his early postoperative days, he developed a subsegmental pulmonary embolism despite being on mechanical venous thromboembolism (VTE) prophylaxis and heparin infusion. However, all other aspects of his postoperative course were uneventful. Postoperative abdominal CT scan was performed with barium injected through the stoma, which showed no leak or collection (Fig. 6). Four weeks following his surgery, he was discharged home in a good condition.

The final histopathology proved the diagnosis of LAMN in all resected specimens. He was regularly followed up in the clinic. His last visit was seven months after surgery, where he appeared well and had no complaints. Closure of the stoma is planned after one year and following reevaluation for any possible recurrence.

## 3 Discussion

The unique challenges of having PMP in an incidentally found malrotation and performing CRS/HIPEC with difficult anastomosis probably make this the first such case to be described anywhere in the world.

Malrotation presents incidentally in adulthood in most cases, either intraoperatively or during investigation for an unrelated disease. Its significance arises when the malrotation poses a difficult or confusing clinical picture that leads to misdiagnosis or delay in management [6,7]. A similar clinical situation occurred in this patient initially during his presentation, and during his CRS with challenging intraoperative resection and anastomosis.

In the literature, the description of PMP or even appendicular mucocele in patients with malrotation is scarce [[8], [9], [10], [11]]. It is very rare to have a combination of two uncommon conditions in the same patient.

To our knowledge, no published paper in the English literature has described CRS/HIPEC for PMP in such a case of malrotation. Orton et al. from the Cleveland Clinic Foundation presented a poster at the Society of American Gastrointestinal and Endoscopic Surgeons (SAGES) meeting in 2011, which described HIPEC for PMP in a patient with situs inversus incompletus. Their patient initially presented with acute appendicitis and was managed with appendectomy. Their CRS did not include extensive colon resection.

In the current case, the division of the colon was at three sites: distal ascending, distal transverse colon, and low rectum. This created a challenging situation in which the remaining part of the colon was dependent on the middle colic vessels. Having a low rectal stump further increased the complexity of the situation.

The creation of colorectal anastomosis can be difficult in several situations. A well-described scenario is when the resection involves most of the left colon and part of the transverse colon, and the remaining transverse colon does not reach the rectal stump. The following techniques have been described to overcome this challenge: Deloyers procedure [[12], [13], [14]]; the retroileal transmesenteric colorectal anastomosis [15,16]. These techniques emphasize the superiority of colorectal anastomosis over the easier-to-perform ileorectal anastomosis. The advantage of colorectal anastomosis is due to its better functional outcome and improved quality of life, particularly the result of reducing the number of bowel motions and other complications through preserving even a small part of the colon.

The case we present here was similarly challenging, though very different from, the scenario we discuss above. In the scenario above managed with the described technical solutions, the remaining colon still has a right colic vascular pedicle. Even in the techniques noted (e.g., Deloyers technique), the middle colic vessels would be divided to allow further mobilization of the right and transverse colon. However, in the patient in our case report, the malrotation and the ileocecal resection and resection of the distal transverse colon down to the low rectum created a segment of transverse colon that is solely supplied by the middle colic pedicle. The patient was young and otherwise healthy, which motivated us even further to find a solution to preserve that part of the colon and provide him a chance of a better functional outcome.

The remaining transverse colon was rotated almost 270° around the middle colic vessels to join the distal transverse colon with the low rectum via a well-vascularized and tension-free anastomosis from the right side. The other end, at the ascending colon, was taken up as a mucous fistula as described above.

This improvised surgical solution, which we now refer to as the Traiki’s technique, allowed the preservation of a good length of healthy colon. This allows the patient the possibility of a better quality of life for the following reasons: 1) avoids diarrhea and its related complications; 2) improves continence due to lower number of bowel movements and preserving colon function as a reservoir. This technique could be utilized in certain operative situations where challenging colorectal resection leaves only part of healthy transverse colon. However, it must be acknowledged that the transverse colon rotation technique (Traiki’s technique) was facilitated in this patient due to the malrotation and the abnormally redundant transverse colon mesentery. His unique anatomy permitted such rotation around the middle colic pedicle without compromising the vascularity or causing tension of the anastomosis.

## 4 Conclusion

• Malrotation can complicate the presentation of appendicular mucocele and cause a significant delay in diagnosis and management. The early recognition and consideration of rare and uncommon situations can save the patient from considerable morbidity.
• When feasible, the preservation of a part of the colon improves quality of life for many patients with complex colon resection. This improvised surgical technique of transverse colon rotation (or Traiki’s technique) is safe and effective in achieving this goal. We hope it provides a solution to some challenging colorectal anastomoses.

## Conflicts of interest

None.

## Sources of funding

No source of funding.

## Ethical approval

Exemption from ethical approval was obtained from IRB at King Saud University, College of Medicine. Project No. E-18-3483.

## Consent

Written informed consent was obtained from the patient for publication of this case report. A copy of the written consent is available for review by the Editor-in-Chief of this journal on request.

## Author’s contribution

Basmah Faris Alhassan: Data acquisition and interpretation, writing and revising of the manuscript.

Abdullah Saji Alharbi: Data acquisition, editing and revising of the manuscript.

Walid Mokhtar Omar: Study design, revising of the manuscript.

Mohammed Ayesh Zayed: Data interpretation.

Maha Abdulla: Editing of the manuscript, data collection.

Thamer Abdullah Bin Traiki: The most responsible physician (MRP). Study concept and design, editing and revising of the manuscript.

All authors have reviewed and approved the final manuscript.

## Registration of research studies

This case report is registered as first-in-man study on www.researchregistry.com website with UIN 4485.

## Guarantor

Thamer Bin Traiki.

Basmah Alhassan.

## Provenance and peer review

Not commissioned externally peer reviewed.
